# The BD Onclarity HPV Assay on Samples Collected in SurePath Medium Meets the International Guidelines for Human Papillomavirus Test Requirements for Cervical Screening

**DOI:** 10.1128/JCM.00508-16

**Published:** 2016-08-24

**Authors:** Ditte Ejegod, Fabio Bottari, Helle Pedersen, Maria Teresa Sandri, Jesper Bonde

**Affiliations:** a Department of Pathology, Copenhagen University Hospital, Hvidovre, Denmark; b Division of Laboratory Medicine, European Institute of Oncology, Milan, Italy; c Clinical Research Centre, Copenhagen University Hospital, Hvidovre, Denmark; Memorial Sloan-Kettering Cancer Center

## Abstract

This study describes a validation of the BD Onclarity HPV (Onclarity) assay using the international guidelines for HPV test requirements for cervical cancer screening of women 30 years old and older using Danish SurePath screening samples. The clinical specificity (0.90, 95% confidence interval [CI] = 0.88 to 0.91) and sensitivity (0.97, 95% CI = 0.87 to 1.0) of the Onclarity assay were shown to be not inferior to the reference assay (specificity, 0.90 [95% CI = 0.88 to 0.92]; sensitivity, 0.98 [95% CI = 0.91 to 1.0]). The intralaboratory reproducibility of Onclarity was 97%, with a lower confidence bound of 96% (kappa value, 0.93). The interlaboratory agreement was 97%, with a lower confidence bound of 95% (kappa value, 0.92). The BD Onclarity HPV assay fulfills all the international guidelines for a new HPV test to be used in primarily screening. This is the first clinical validation of a new HPV assay using SurePath screening samples, and thus the Onclarity HPV assay is the first HPV assay to hold an international validation for both SurePath and ThinPrep.

## INTRODUCTION

The increased evidence for use of molecular human papillomavirus (HPV) testing to detect cervical cancer precursors and cancers in screening has resulted in a surge of commercial molecular HPV tests. Defined clinical standards for the performance of HPV tests have been set forth in *Guidelines for Human Papillomavirus DNA Test Requirements for Primary Cervical Cancer Screening in Women 30 Years and Older* (1). The clinical standards are based upon data from four European large prospective randomized screening trials (2–6). The international standards ensure that the performance of new HPV tests is not inferior to the HPV assays used in randomized clinical trials. However, only a select few assays have obtained the clinical validation in accordance with the international guidelines (7). For validation purposes, the HPV assay to be evaluated and sample collection methodology are both important. Today, molecular HPV testing for screening purposes is often performed on liquid-based cytology (LBC) samples, which in contrast to conventional pap smears can be used for both primary cytology screening with HPV triage and HPV screening with cytology triage. Currently, two LBC collection media dominate the field of cervical screening: Hologic ThinPrep and BD SurePath. The majority of internationally validated HPV assays for cervical cancer screening have obtained the validation on ThinPrep-collected samples (8–15), including the Onclarity HPV assay (10). One assay has obtained the validation using SurePath media (16). The Onclarity assay is a HPV DNA real-time PCR targeting the viral genes E6 and E7 of the 13 high-risk (hr) HPV genotypes and HPV66 (9, 10, 17–19). The Onclarity assay includes extended genotyping, allowing individual detection of the six genotypes HPV16, -18, -31, -45, -51, and -52, with an additional eight genotypes detected in three distinct groups: HPV33/58, HPV56/59/66, and HPV35/39/68. The assay also incorporates a beta-globin internal control for sample sufficiency and assay performance.

We present validation data on Onclarity with SurePath-collected samples in accordance with the international criteria for use in primary HPV screening. Test performance was compared to Hybrid Capture 2 (HC2) for clinical specificity and sensitivity on samples from Danish women undergoing screening and follow-up in the organized Danish Screening program. This validation has the added impact of being the first validation of a commercial HPV assay on SurePath-collected cytology samples. SurePath is the predominantly used cytology medium in the Danish screening program, accounting for ca. 85% of the 450,000 routine cervical cancer screening samples taken annually.

## MATERIALS AND METHODS

### Sample collection.

For the specificity analysis (the control population), residual SurePath (BD Diagnostics [BD], Burlington, NC) material from 2,840 routine consecutive samples were collected from Danish women ≥30 years old undergoing routine cervical cancer screening at Copenhagen University Hospital, Hvidovre between April and September 2014. After the samples had been collected, we excluded 1,189 samples due to insufficient SurePath material (defined as <1.6 ml postcytology) to ensure enough material for all testing needs under the study protocol.

An initial pool of 1,270 samples from 1,651 eligible women were screened with both HC2 (Qiagen, Hilden, Germany) and Onclarity (BD). A review of the complete screening history of all 1,270 women from January 2000 to November 2015 in the Danish National Pathology Databank resulted in the following exclusions: 103 samples were excluded since the patients had a history of cytological diagnosis of atypical squamous cells of undetermined significance (ASCUS) in the past 15 months, cytological diagnosis of ASCUS or higher-grade lesions (≥ASCUS) in the past 12 months, or previous cervical cancer or cervical intraepithelial neoplasia (CIN) in the previous 3 years. Thirteen additional samples from women with ≥CIN2 on follow-up were also excluded. In total 1,154 <CIN2 samples were included in the Control population (median age, 43 years; range, 30 to 80 years; interquartile range [IQR], 37 to 52 years). For the sensitivity analysis (the case population), residual SurePath material from 411 consecutive, unselected samples was collected from women undergoing cervical cancer screening at Copenhagen University Hospital, Hvidovre, Denmark, between September and October 2012 within the population-based screening setting in the Capital Region of Denmark. The samples were derived from women diagnosed with histologically confirmed CIN2+ detected by cervical screening on the basis of an abnormal liquid-based cytology (≥ASCUS) and/or a positive hrHPV HC2 result. Subsequent to the collection, 10 samples with insufficient quantity of residual SurePath material postcytology were excluded (defined as samples with ≤1.0 ml of total residual material). All 401 included samples were tested using Onclarity, HC2, and CLART HPV2. Follow-up histology results were retrieved from the Danish National Pathology databank. This revealed a total of 61 samples from women above the age of 30 years with confirmed ≥CIN2 histology, and these constituted the case population (median age, 36 years; range, 30 to 73 years; IQR, 32 to 46.5 years).

For assay reproducibility assessment, 865 unselected, deidentified residual SurePath samples were collected. A total of 500 consecutive samples were included in the analysis; of these, 156 were determined to be positive for hrHPV by HC2. Three aliquots of the residual vial SurePath sample were independently tested with the Onclarity assay. The first two aliquots were used for intralaboratory reproducibility (at Copenhagen University Hospital), and the last aliquot was used to assess interlaboratory agreement (at the European Institute of Oncology, Milan, Italy). Reproducibility testing was conducted by the same staff in each location and was within the manufacturer-defined material stability claim (30 days at 2 to 30°C).

### HPV testing. (i) BD Onclarity HPV assay.

Residual SurePath samples were tested with the Onclarity assay as previously described (18). In summary, 0.5-ml aliquots of residual vial SurePath material were transferred into a BD HPV LBC diluent medium tube (BD), inverted three to four times prior to preheat treatment for 30 min at 120°C on a BD prewarming station. The prewarmed samples were tested on the automated Viper LT platform according to manufacturer recommendations. The Viper LT platform conducts 30 samples per run, with an integrated work-flow where DNA extraction, template pipetting to PCR master mix, and real-time PCR are fully automated. The hands-on working time per batch is ∼20 min, including daily maintenance. A full work flow from loading to results consists of 4.5 h of hands-free work time. However, the instrumentation allows for overlapping batch runs, meaning that one instrument processes and returns results on 90 samples per working day (Danish standard 7.5-h work day) or 120 samples for extended work days (Danish standard for extended clinical operations 10 h/day).

### (ii) HC2.

Hybrid Capture 2 (HC2; Qiagen, Hilden, Germany) analysis was done on the resuspended postcytology pelleted SurePath material (postquot material) from the cytology procedure as specified by the manufacturer. All samples were denatured manually prior to HC2 analysis. The control samples were tested using the automated Rapid Capture System (Qiagen, Germantown, MA), and the case samples were tested on the manual HC2 modular system (Qiagen, Germantown, MA). All testing was performed according to the manufacturer's specifications. No retest range (equivocal zone) was used.

### (iii) CLART HPV2 full genotyping assay.

A 0.5-ml portion of residual vial SurePath material was used for DNA purification (MagnaPure LC total nucleic acid isolation kit; Roche, Rotkreutz, Switzerland) and subsequent CLART HPV2 analysis (Genomica, Madrid, Spain), as previously described (20). In summary, SurePath material was spun down, treated with proteinase K for 1 h at 56°C, followed by 1 h at 90°C to reverse formaldehyde-induced cross-linking. PCR amplification was performed using a CLART HPV2 amplification kit (Genomica), and visualization was performed according to the manufacturer's specifications. The genotyping results were analyzed and reported automatically on the clinical array reader (Genomica).

### Ethical approval.

The case population was collected within the Danish arm of the European CE-IVD evaluation of the BD Onclarity HPV assay. The study was approved by the Danish regional ethical committee (Ethical committee protocol H4-2012-070; ClinicalTrials.Gov, ID NCT0671462).

The samples comprising the control and reproducibility populations were undertaken on residual routine samples, which would otherwise have been discarded, as a quality development study. In Denmark, such studies do not require ethical approval. The Danish Data Inspection Agency (AHH-2015-087/04154) was notified prior to initiating the study.

### Statistical analysis.

A positive HPV test was defined according to the manufacturer's recommendations (the HC2 relative light unit per cut off was ≥1; *C_T_* values were ≤34.2 for all Onclarity individual channels and internal controls). CLART was used as full genotyping assay, and all 35 genotypes were reported. Clinical specificity and sensitivity values for Onclarity were compared to those for HC2 using the noninferior score test, where noninferiority is defined as a relative specificity for <CIN2 of ≥98% and a relative sensitivity for ≥CIN2 of ≥90% (1). For the intralaboratory reproducibility and interlaboratory agreement, a lower confidence bound of ≥87% was used as a threshold (1). The excel sheet provided by VU University Medical Centre Amsterdam (original design by Johannes Berkhof) was used for the noninferiority score test. For other statistical computations, SPSS statistics 22 software was used.

## RESULTS

### Clinical specificity and sensitivity analyses.

For the evaluation of specificity, valid results on Onclarity and HC2 were obtained on a total of 1154 SurePath samples from women 30 years old and older (median age, 43 years; range, 30 to 86 years) with <CIN2 follow-up, undergoing routine primary cervical cancer screening. The clinical specificity of Onclarity was 0.90 (95% CI = 0.88 to 0.91), which was similar to that of HC2 (0.90; 95% CI = 0.88 to 0.92) (Table 1). The clinical specificity of Onclarity was not inferior to that of HC2 (*P* = 0.02, Tables 1 and 2).

**TABLE 1:** BD Onclarity HPV findings among 1,154 primary screening samples without ≥CIN2 follow-up and 61 follow-up samples with confirmed ≥CIN2 in relation to HC2

Sample type and Onclarity assay result^*a*^	HC2 result (no.)		Total no.
	HrHPV positive	HrHPV negative	
Control (<CIN2)			
HrHPV positive	90	30	120
HrHPV negative	26	1,008	1,034
Total	116	1,038	1,154
Case (≥CIN2)			
HrHPV positive	58	1	59
HrHPV negative	2	0	2
Total	60	1	61

a Control (<CIN2): test statistic (T) = 2.12 and *P* = 0.02. Case (≥CIN2): test statistic (T) = 1.97 and *P* = 0.02.

**TABLE 2:** Clinical specificity and sensitivity for BD Onclarity HPV and HC2 assays

Specificity or sensitivity (CIN status)	Specificity and sensitivity (range)	
	Onclarity	HC2
Specificity (<CIN2)	0.90 (0.88–0.91)	0.90 (0.88–0.92)
Relative specificity (<CIN2)	1.0 (0.97–1.02)	1.0
Sensitivity (≥CIN2)	0.97 (0.87–1.0)	0.98 (0.91–1.0)
Relative sensitivity (≥CIN2)	0.98 (0.93–1.04)	1.0

For clinical sensitivity analysis, a total of 61 SurePath samples with valid results on Onclarity and HC2 were used. The samples were from women 30 years old and older (median age, 36 years; range, 30 to 73 years) with confirmed ≥CIN2 histology: 17 with CIN2, 41 with CIN3, and 3 with cervical cancer. The clinical sensitivity of Onclarity was 0.97 (95% CI = 0.87 to 1.0) for ≥CIN2. For comparison, the sensitivity for HC2 was 0.98 (95% CI = 0.91 to 1.0). Overall, the clinical sensitivity of Onclarity was not inferior to that of HC2 (*P* = 0.02, Tables 1 and 2). Two samples were determined to be negative by Onclarity and hrHPV positive by HC2, a CIN2 and a CIN3 case, respectively. Genotyping by CLART HPV2 using modified L1 PGMY 09/11 primers showed these two specimens to be positive for non-high-risk HPV genotypes 70 and 82, respectively (Table 3). In addition, one sample was negative by HC2 but HPV 16 by Onclarity. This sample was HPV negative by CLART HPV2 but the histology result was confirmed as a CIN2 (Table 3).

**TABLE 3:** Detailed results of the Onclarity and HC2 discordant samples from women with confirmed ≥CIN2

Sample	Test result			
	HC2	Onclarity	CLART HPV2	Histology diagnosis
1	Negative	16^*a*^	Negative	CIN2
2	Positive	Negative	82^*a*^	CIN3
3	Positive	Negative	70^*a*^	CIN2

a HPV genotype for which the sample tested positive.

### Intralaboratory reproducibility and interlaboratory agreement.

The intralaboratory reproducibility and interlaboratory agreements were assessed by using a set of 500 samples, including 156 determined to be hrHPV positive by HC2 (31%). The samples were split in three aliquots, with the first two aliquots used for intralaboratory reproducibility (Copenhagen laboratory results 1 and 2). The third aliquot was send to Milan, Italy, for interlaboratory agreement (Milan laboratory result). All included samples had a valid HC2 and an Onclarity result on all three runs. The intralaboratory reproducibility was found to be 97.4% (lower confidence bound = 95.9% and kappa value = 0.93). The positive and negative reproducibilities were 92.9 and 99.2%, respectively. The interlaboratory agreement was 96.8% (lower confidence bound = 95.2% and kappa value = 0.92) (Table 4). The reproducibility of the individual genotype results showed good agreement, with an average kappa value of 0.905 (range, 0.78 to 1.0) for all nine genotype groups detected by the assay design (Table 5).

**TABLE 4:** Intralaboratory reproducibility and interlaboratory agreement of the BD Onclarity HPV assay using SurePath screening samples

Intralaboratory reproducibility or interlaboratory agreement^*a*^	HrHPV status	Copenhagen laboratory result 1 (no. of samples)		Total no.
		HrHPV positive	HrHPV negative	
Reproducibility				
Copenhagen laboratory result 2	HrHPV positive	130	3	133
	HrHPV negative	10	357	367
	Total	140	360	500
Agreement				
Milan laboratory result	HrHPV positive	133	9	142
	HrHPV negative	7	351	358
	Total	140	360	500

a The intralaboratory reproducibility was 97.4% (lower confidence bound, 95.9%; kappa value, 0.93). The interlaboratory agreement was 96.8% (lower confidence bound, 95.2%; kappa value, 0.92).

**TABLE 5:** Intralaboratory reproducibility and interlaboratory agreement of genotype findings

Assessment and HrHPV type	No. of genotype findings per run or per laboratory^*a*^			No. negative for both runs	Kappa value	95% CI^*b*^	
	Combined result	First run	Second run			Lower	Upper
Intralaboratory reproducibility							
16	22	6	0	472	0.87	0.77	0.97
18	9	4	1	486	0.78	0.59	0.97
31	17	0	3	480	0.92	0.82	1.00
45	11	0	0	489	1.00	1.00	1.00
51	9	1	3	487	0.81	0.64	0.99
52	19	5	1	475	0.86	0.75	0.97
33/58	16	1	1	482	0.94	0.86	1.00
56/59/66	29	1	1	469	0.97	0.92	1.00
35/39/68	25	2	0	473	0.96	0.90	1.00
Interlaboratory agreement							
16	24	4	0	472	0.92	0.84	1.00
18	11	2	1	486	0.88	0.74	1.00
31	16	1	2	481	0.91	0.81	1.00
45	10	1	0	489	0.95	0.86	1.00
51	10	0	3	487	0.87	0.72	1.00
52	20	4	1	475	0.88	0.78	0.99
33/58	15	2	1	482	0.91	0.80	1.00
56/59/66	28	2	4	466	0.90	0.82	0.98
35/39/68	25	2	0	473	0.96	0.90	1.00

a The number of genotype findings per run is specified for the intralaboratory reproducibility data; the number of genotype findings by laboratory is specified for the interlaboratory agreement data.

b CI, confidence interval.

## DISCUSSION

In this study we compared the clinical performance of the BD Onclarity HPV assay to that of HC2 on SurePath-collected samples from Danish women undergoing cervical cancer screening. The clinical specificity and sensitivity of Onclarity was found to be not inferior to that of HC2 using the internationally defined thresholds of 98 and 90%, respectively (1). The Onclarity assay displayed high intralaboratory reproducibility and interlaboratory agreement with both lower confidence bounds of reproducibility and agreement higher than the recommended 87%. The corresponding kappa values were >0.9 for both intra- and interlaboratory comparisons (1). The reproducibility of the genotype findings displayed an average kappa value of 0.905, indicating a solid assay performance. The latter is equally important since the overall reproducibility from the perspective that if genotype information is to be used for risk stratification of the individual woman, confidence in an assays ability to reproduce a genotype finding will be pivotal to the clinical performance of such a strategy.

Among the case samples, three showed discordant HC2 and Onclarity results. Of these three samples, one with confirmed CIN2 histology tested negative using HC2 but was determined to be HPV16 using Onclarity. Subsequent genotyping of the sample by CLART HPV2 proved negative. The discrepancy between CLART HPV2 and Onclarity could be due to PCR amplicon length and/or differences in the molecular target gene: Onclarity has an approximately 165-bp amplicon from E6 and E7, whereas CLART HPV2 amplifies ∼465 bp from the HPV L1 gene. Alternatively, the L1 target gene could have been deleted as a result of virus integration. Two samples were determined to be negative using Onclarity but positive using HC2. The CLART HPV2 reported non-high-risk HPV genotypes 82 and 70, respectively; both genotypes are generally recognized as cross-reacting in the HC2 assay (21–24). Despite the histology diagnosis of CIN3 and CIN2, respectively, genotypes 70 and 82 are rarely the cause of invasive cervical cancer (25).

In the present study, we used the HC2 HPV assay as the comparator test. The HC2 assay has been thoroughly clinically validated (1) and has been extensively used in other studies for validation of new HPV assays (8, 10, 11, 16).

This study is the first to use the international guidelines to validate a new commercial available HPV assay using screening samples collected in SurePath medium. SurePath is a cytology sample collection medium where a low concentration of formaldehyde is added to the alcohol fixative to ensure adequate preservation of the cell material for cytology. The adequacy of SurePath-collected samples for molecular HPV analysis has been questioned due to the ability of formalin to cross-link DNA and protein (26–28). However, Agreda et al. reported no deterioration in performance in SurePath specimens stored over 2.5 years (29). These data, along with our previous Onclarity studies (18) and published results from the Danish Horizon study (30–37), show that SurePath is indeed a suitable sample collection media for molecular HPV testing. Moreover, SurePath-collected cytology samples also provide a better cytology quality with fewer inadequate cytology results than ThinPrep and overall provides a higher quality LBC screening, detecting more CIN2+ disease (38, 39). Until primary HPV screening is fully implemented, cervical screening is reliant on high-quality LBC cytology and HPV for triage in combination. The suitability of a sample collection medium that allows for both high-quality cytology and HPV testing on the same sample is pivotal to a high-performance screening program as the organized Danish cervical cancer screening.

For SurePath using laboratories with evidence based clinical practice approach, international validation of a HPV assay on SurePath taken samples is important, since all previously international HPV assay validations are made almost exclusively on ThinPrep-obtained samples. To this end, it is encouraging that Onclarity has also previously been evaluated on ThinPrep-collected samples (9, 10, 17, 19, 21, 40–42). Two of these studies using ThinPrep-collected samples have used the international consortium guidelines for validation (9, 10). In our previous study, we used HC2 as a comparator assay, whereas Cuschieri and coworkers used GP5+/GP6+ as a comparator assay. The former study was performed partly by our lab using screening samples from the United Kingdom (the Predictor Study cohort) for the clinical validation part and concluded that the use of Onclarity on ThinPrep samples was not inferior to HC2 for both clinical specificity and sensitivity (10). The Scottish Onclarity study (9), however, used screening samples from Scottish women with the VALGENT adaption of the International guidelines. Here, it was found that although the clinical sensitivity of Onclarity was not inferior to that of G5+/GP6+, the clinical specificity was not. Cuschieri et al. speculated that the high prevalence (18%) in Scotland is causing a challenge for the specificity criterion. Moreover, the Scottish study also pointed out that samples from women below the age of 30 were included, which notoriously is an age group where many clinically insignificant and transient infections are observed. Thus, this study was not fully compliant with the specificity criteria as defined by Meijer et al. (1). The present study, as well as the Denmark/United Kingdom study (10), included only women 30 years old and older. In conclusion, the Onclarity assay is the first commercial HPV assay to obtain international guideline validation on both samples collected in ThinPrep and SurePath, thereby allowing an evidence-based choice of this HPV assay without regard to the LBC medium used for collecting the routine cervical screening samples.

The Onclarity has extended genotyping with individual genotyping of six genotypes (16, 18, 31, 43) and the remaining eight genotypes in three distinct groups (HPV33/58, HPV56/59/66, and HPV35/39/68). With the exception of separate follow-up guidelines in many countries for HPV16 and/or HPV18 in cotesting, triage (49; Rijksinstitute voor Volkgezondheid [http://www.rivm.nl/bevolkingsonderzoeknaarkanker]), or primary screening (43, 49), the knowledge that different hrHPV genotypes confers different risks (44–46) has not yet been transformed into clinically distinct guidelines. Schiffman et al. (47) used the Onclarity assay to investigate whether genotyping could be used to manage women with ASCUS and HPV. These authors found a distinction in the risk potential between the different genotypes detected by Onclarity indicating that ultimately genotyping could be used to risk stratify women for follow-up, hopefully reducing overtreatment after an HPV-positive screening sample. However, further studies on risk stratification by genotyping are needed. The Onclarity assay can be run on the fully automated Viper LT Platform, which can be used for running both LBC samples and paraffin-embedded formalin-fixed cervical screening samples (48), making it a very versatile assay.

In conclusion, our data indicate that the Onclarity HPV assay performs clinically comparably to HC2 and meets the cross-sectional guidelines for HPV test requirements for primarily screening for specificity and sensitivity for ≥CIN2 and inter- and intralaboratory reproducibility. In addition, the Onclarity assay has the added benefit of extended genotyping and automated workflow that can be used regardless of LBC collection media. Our study shows that Onclarity performs at the highest internationally defined level with respect to comparator assays on SurePath-collected samples.
